# Massive Presentation of a Neglected Basal Cell Ulcer on the Forehead and Outcome With Radiation Therapy: A Case Report and Literature Review

**DOI:** 10.7759/cureus.34383

**Published:** 2023-01-30

**Authors:** Sindu Iska, Gerald Sokol, Ali Sawani, Prateek Patibandla

**Affiliations:** 1 Internal Medicine, Cleveland Clinic Florida, Weston, USA; 2 Radiation Oncology, Uniformed Services University of the Health Sciences, Bethesda, USA; 3 Internal Medicine, Cleveland Veterans Affairs Medical Center, Cleveland, USA; 4 Hospital Medicine, Presbyterian Hospital, Albuquerque, USA

**Keywords:** basal cell carcinoma of scalp, radiation therapy, seizures, neglected, aggressive

## Abstract

Basal cell carcinoma (BCC) of the scalp is the most common cancer of the skin and is locally invasive. The patched/hedgehog intracellular signaling pathway is responsible for regulating cell growth and tumor formation by inactivating mutation of protein patched homolog 1 (PTCH1) or activating mutation of Smoothened (SMOm). BCC can cause significant morbidity from local destruction if neglected. The risk of metastasis and death is 6.5% in tumors greater than or equal to 2 cm in size. The gold standard treatment is surgical excision. Radiation therapy is used to treat skin cancers as an adjuvant or in patients who are not candidates for surgical intervention or who refuse therapy. It works by using low-energy X-rays or electron beam radiation. They work on the superficial skin and do not affect the organs deeper. Here, we describe the case of a man who presented with an unwitnessed seizure and was found to have a large ulcer on his forehead, which was later diagnosed to be BCC of the scalp eroding the calvarium. The base of the ulcer was the patient's dura and brain. He was successfully treated with electron beam radiation therapy for six weeks with careful preservation of brain tissue. The patient’s skin was re-epithelialized and the bone was recalcified. The ulcer on the forehead has completely regressed. This case report and literature review illustrates the evidence to propose the importance of radiation therapy and its potential to be the first-line treatment in BCC, especially in similar cases like ours. Multimodality treatment with a radiation oncologist, dermatologist, and medical oncologist can save patients from devastating outcomes.

## Introduction

Basal cell carcinoma (BCC) is the most common cancer in Caucasians, with the incidence rising by 4%-8% annually [1]. The patched/hedgehog intracellular signaling pathway regulates cell growth and tumor formation by inactivating mutation of protein patched homolog 1 (PTCH1) or activating mutation of Smoothened (SMOm) [2]. BCC can cause significant morbidity from local destruction if neglected. The risk of metastasis and death is 6.5% in tumors greater than or equal to 2 cm in size [3]. Radiation therapy (RT) causes damage either directly by interacting with cellular DNA or indirectly by creating free radicals derived from the ionization or excitation of the water component of cells. Reactive oxygen species (ROS) can lead to mitochondrial damage and DNA mutations and act as signaling molecules to enhance essential growth and proliferation pathways, including PI3K and hypoxia-inducible factors. RT aims to prevent cancer cells from multiplying and damaging the DNA beyond repair [4]. Few studies have emphasized the concurrent use of RT, and hedgehog inhibitors have better outcomes, especially in recurrent advanced BCC [5]. RT achieves its therapeutic effect by inducing different types of cell death through apoptosis, mitotic catastrophe, necrosis, senescence, and autophagy [6]. Through this case, we would like to emphasize the impact of RT on massive BCC like our patient.

## Case presentation

**Figure 1:**
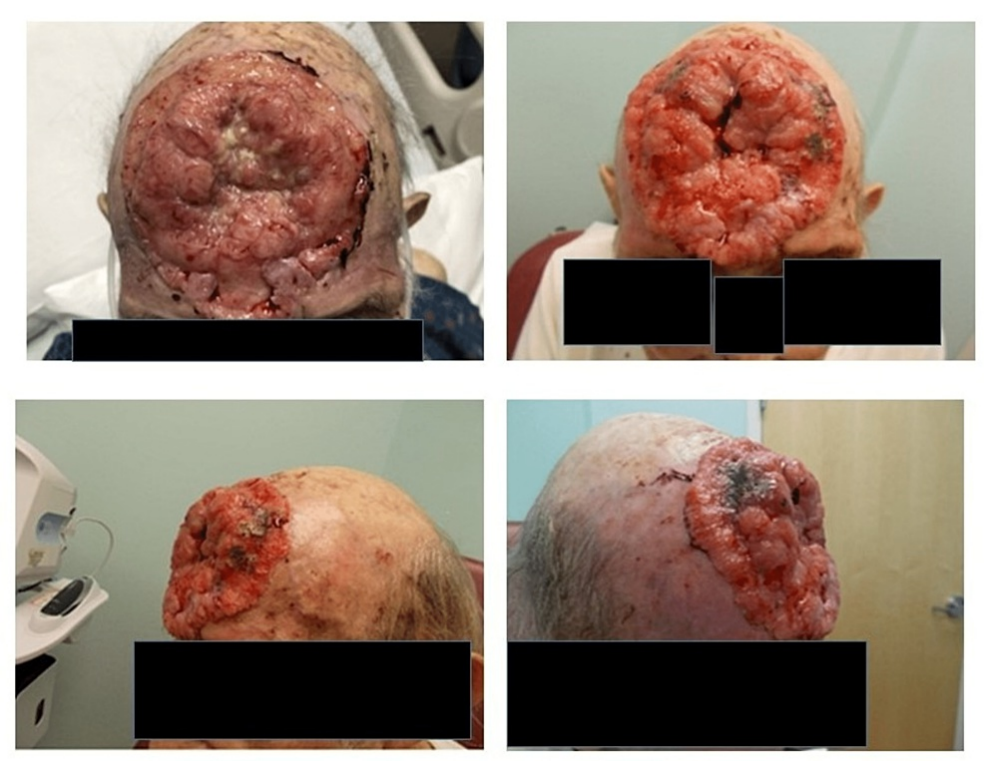
Forehead ulcer on presentation. A large fungating ulcer of approximately 9 x 10 cm with pinpoint bleeding sites and rolled-out borders on the forehead.

A 76-year-old Caucasian male landscaper with no known past medical history except for smoking (61 pack-years) presented to the emergency department. He was brought in after a motor vehicle accident (MVA) for medical clearance. The patient had not visited a physician by choice in the past 35 years, and his daughter at the bedside reported her concern regarding a chronic forehead wound. He had a small lesion a few years ago and covered it with a beanie. The patient was hesitant to reveal his wound, and after persistent persuasion, he revealed that his wound started as a pea-sized lesion seven years ago and has been progressive since. He complained of occasional fatigue and bleeding from the forehead lesion. The patient denied headaches, seizures, blurring of vision, weight changes, change in appetite, abdominal pain, nausea, vomiting, chest pain, shortness of breath, neurological deficits, focal deficits, and incontinence. On physical examination, he appeared to be a cachectic, disheveled male. A cardiac exam revealed a grade 2 systolic murmur in the mitral area. As shown in Figure 1, a skin exam showed an approximately 9 x 10 cm fungating ulcer on the forehead with pinpoint bleeding sites and rolled-out borders.

**Figure 2:**
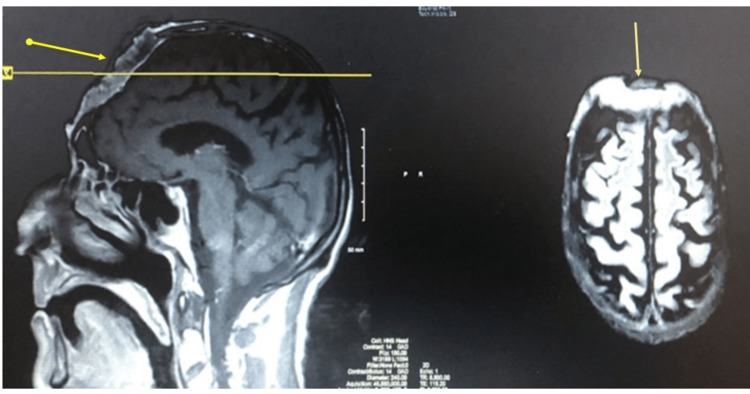
MRI skull and brain of the patient on presentation. The arrows show the eroding lesion through the skull into the dura. MRI: magnetic resonance imaging.

The rest of his exam was unremarkable. His labs showed hemoglobin 4 g/dl, MCV 66, and his stool occult blood was positive. As shown in Figure 2, our patient's imaging showed a full-thickness erosion of the frontal bone with likely invasion into the dural layer. 

**Figure 3:**
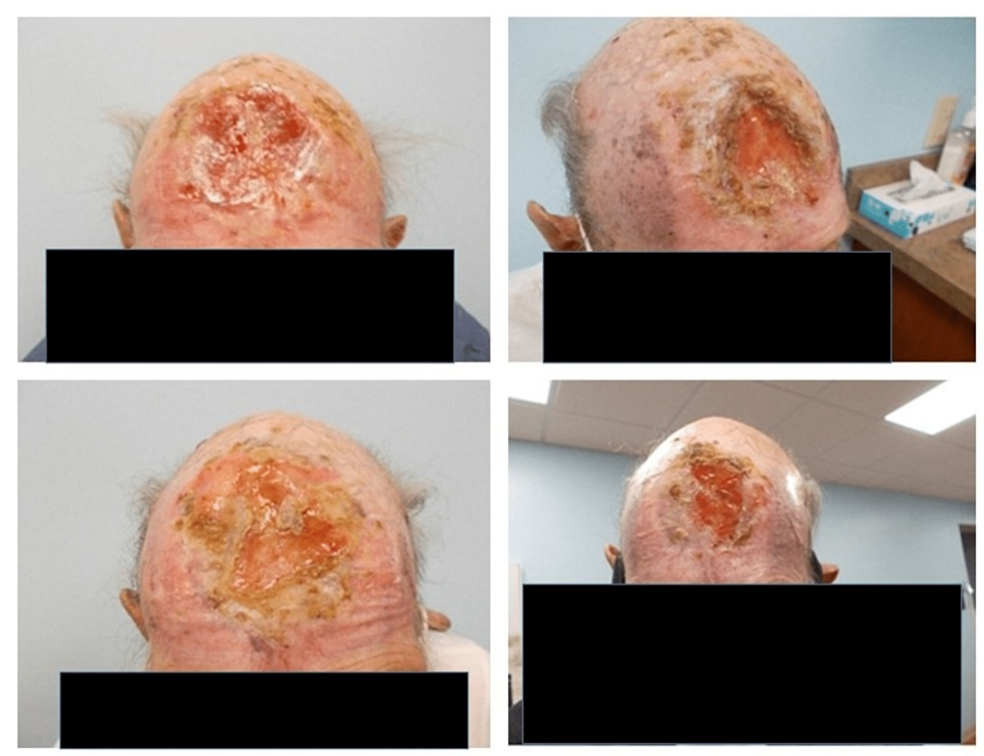
Regression of the forehead ulcer after RT. The upper image is on day 92, measuring approximately 5 cm x 4.5 cm. The lower left image is on day 183, measuring approximately 3 cm x 3.5 cm. The lower right image is on day 355 during his last follow-up visit after RT, measuring approximately 2 cm x 1.5 cm. RT: radiation therapy.

His MVA was attributed to a possible seizure secondary to the forehead ulcer base on the dura, he was placed on seizure prophylaxis, and oncology was consulted and recommended a punch biopsy. A 4 mm punch biopsy of this mass was consistent with nodular BCC. The patient left the hospital against medical advice prior to his gastroenterology workup for his anemia with his complete medical workup. Due to the patient’s questionable compliance over the last few decades regarding his personal health, after extensive counseling, he agreed to an oncology follow-up. Fortunately, he followed up, and his positron emission tomography (PET) scan revealed a large multilobulated soft tissue mass along the right frontal scalp causing bony destruction extending to the inner table of the frontal bone. The maximum standardized uptake value (SUV) was 9.5. A multidisciplinary team reviewed his clinical scenario and agreed on local beam radiotherapy, as he was not a candidate for surgery. He was successfully treated with electron beam RT at depth for 30 fractions over six weeks, with special consideration given to carefully preserve his brain tissue due to his proximity to the lesion. The patient's skin was re-epithelialized and the bone was recalcified after his RT. In the span of his one-year follow-up, the ulcer on the forehead regressed, as shown in Figure 3. Unfortunately, the patient was lost to follow-up after a year.

## Discussion

BCC of the scalp and calvarium with intracranial extension and dural involvement is rare; however, it could show aggressive behavior if neglected [7]. Only 1% of giant BCCs attain a size larger than 5 cm, and one-third of the 1% is due to neglect in seeking medical advice, as in our patient. The most common reasons are low social milieu/lack of insurance, old age, slow growth, and painless ulcer delay in the care required. Local treatment is preferred due to its low metastatic potential. RT is just as effective in treating aggressive histologic tumors and in patients where surgery is contraindicated or unresectable [8]. RT has shown an excellent effect on the nodular subtype. Surgical options like Mohs micrographic surgery are the treatment of choice for high-risk and recurrent BCC with or without electron beam RT. Systemic agents that inhibit the hedgehog pathway, such as vismodegib, are helpful palliative agents for advanced BCC [9]. The treatment of choice is often surgery alone or combined with radiotherapy. Generally, RT for a BCC lesion consists of daily fractions of 1.5-3 Gy given over several weeks. RT causes damage either directly by interacting with cellular DNA or indirectly by creating free radicals derived from the ionization or excitation of the water component of cells. RT aims to prevent cancer cells from multiplying and damaging the DNA beyond repair [4]. RT achieves its therapeutic effect by inducing different types of cell death through apoptosis, mitotic catastrophe, necrosis, senescence, and autophagy [6]. Cure rates have not typically been assessed histologically through RT. However, the visible clinical improvement of the patient’s skin has been unremarkable. We were unable to do a repeat brain scan or biopsy as he was lost to follow-up. We would like to emphasize that RT can be used to convert an inoperable skin lesion into an operable lesion. In the treatment of BCC of the face of less than 4 cm in diameter, surgery should be preferred to radiotherapy. Morphea-type BCCs can be cured with RT; therefore, X-rays have a place in the armamentarium of therapeutic modalities for this tumor if surgery is not feasible or if the patient refuses it. Post-radiation adverse events include acute radiation-related skin toxicity, potential radiation-related changes to underlying structures, and the increased difficulty of managing recurrences within the radiation field. Late adverse events can result in alopecia, cartilage necrosis, and pigmentary skin changes, in addition to the risk for secondary malignancy [10]. However, the benefits outweigh the adverse effects regarding healing, painless mode of treatment, and control of bleeding and infection compared to surgery. RT is also known to have a systemic anticancer response known as the abscopal effect and could be evaluated for future treatment in skin cancer and other organ cancers [11].

## Conclusions

RT is an effective tool for the treatment of cancer due to its systemic anticancer response, known as the abscopal effect. Despite the choice of the best possible treatment modalities, it is not uncommon to encounter a giant neglected skin cancer in the 21st century. Our patient’s skin was re-epithelialized and the bone was recalcified. The ulcer on the forehead has slowly regressed even after completing RT. This case report emphasizes the use of RT as the sole treatment for BCC with excellent results and prognosis by carefully marking the margins with the dermatoscope and precisely using RT to reduce the recurrence of BCC and making it as effective as surgery.
